# Assessing anticancer, antidiabetic, and antioxidant capacities in green-synthesized zinc oxide nanoparticles and solvent-based plant extracts

**DOI:** 10.1016/j.heliyon.2024.e34073

**Published:** 2024-07-04

**Authors:** Muhammad Azeem, Muhammad Hussnain Siddique, Muhammad Imran, Muhammad Zubair, Rabia Mumtaz, Madiha Younas, Mostafa A. Abdel-Maksoud, Mohamed A. El-Tayeb, Muhammad Rizwan, Jean Wan Hong Yong

**Affiliations:** a Department of Bioinformatics and Biotechnology, Government College University Faisalabad, Faisalabad, 38000, Pakistan; b Department of Environmental Sciences, COMSATS University Islamabad, Vehari-Campus, Vehari, 61100, Pakistan; c Gdansk University of Technology, Faculty of Civil and Environmental Engineering, Department of Sanitary Engineering, 80 – 233, Gdansk, Poland; d Botany and Microbiology Department, College of Science, King Saud University, Saudi Arabia; e Department of Environmental Sciences, Government College University Faisalabad, Faisalabad, 38000, Pakistan; f Department of Biosystems and Technology, Swedish University of Agricultural Sciences, 23456, Alnarp, Sweden

**Keywords:** *Acacia nilotica*, Liver cancer, HepG2, α-Glucosidase inhibition assay, DPPH assay

## Abstract

Cancer and diabetes represent significant challenges in the field of biomedicine, with major and global impacts on public health. *Acacia nilotica*, commonly called 'gum arabic tree,' is recognized for its unique biomedical properties. The current study aimed to investigate the pharmacological potential of *A. nilotica*-based zinc-oxide nanoparticles (ZnO-NPs) in comparison to the ethanol and methanol-based extracts against cancer, diabetes, and oxidative stress. Green synthesis of ZnO-NPs was performed using barks of *Acacia nilotica*. Different techniques for the characterization of ZnO-NPs, including UV–Visible spectroscopy, Scanning Electron Microscopy, Fourier Transmission Infrared (FT-IR) spectroscopy, and X-ray Diffraction (XRD), were utilized. The morphological analysis of ZnO-NPs revealed that the fine NPs have mean particle sizes of 15 ± 1.5 nm. For the solvent based-extraction, leaves and barks were utilized and dissolved into ethanol and methanol for further processing. The MTT assay revealed that the optimum concentration of ZnO-NPs to inhibit the proliferation of liver cancer cell line HepG2 was 100 μg/mL where 67.0 % inhibition was observed; and both ethanol- and methanol-based extracts showed optimum inhibition at 100 μg/mL. The DPPH assay further demonstrated that 250 μg/mL of ZnO-NPs and 1000 μg/mL of both ethanol- and methanol-based extracts, as the optimum concentration for antioxidant activity (with 73.1 %, 68.9 % and 68.2 % inhibition respectively). The α-Glucosidase inhibition assay revealed that 250 μg/mL of ZnO-NPs and 10 μg/mL of both ethanol- and methanol-based extracts as the optimum concentration for antidiabetic activity (with 95 %, 93.7 % and 93.4 % inhibition respectively). The study provided interesting insights into the efficacy and reliability of ZnO-NPs for potential pharmacological application. Further research should be focused on examining specific pathways and the safety of ZnO-NPs in comparison to solvent-based extracts.

## Highlights

• Green ZnO NPs (nanoparticles) and solvent-based plant extracts were evaluated for their potential anticancer, antidiabetic, and antioxidative properties.
• NPs were characterized with UV–visible spectroscopy, XRD, and SEM.
• The average size of NPs was 15 ± 1.5 with triangular shape and crystalline forms.
• NPs showed better anticancer, antidiabetic, and antioxidative potential than the solvent-based extracts.

## 1 Introduction

Cancer and diabetes represent significant challenges in the field of biomedicine, with major and global impacts on public health [1,2]. The frequency of cancer and diabetes cases has been increasing which necessitates immediate attention towards finding better therapies and early detection protocols. Liver cancer has been one of the major reasons for medical treatments and death in developing countries [1]. Diabetes, on the other hand, is defined by Refs. [[3], [4], [5], [6]], as a metabolic disorder accompanied by elevated blood glucose levels for prolonged periods insufficiently utilized by the body, leading to severe complications including cardiovascular diseases, ketoacidosis, renal failure, stroke, foot ulcers, and retinal damage. Another complication that is closely linked to diabetes and cancer is oxidative stress [7,8]. Oxidative stress is ascribed to reactive oxygen species (ROS) production. Whenever the anti-oxidant capacity of a cell is reached, these reactive oxygen species cause oxidative stress that leads to cancer cell death [9]. As per reported by the World Health Organization (WHO), approximately 60 % of the world's population relies on conventional therapeutic strategies for most of their basic healthcare needs. In addition, 80 % of the developing world also relies on folk medicine to treat different ailments. To cope with the shortcomings in healthcare systems of the lesser developed countries, phytochemicals and nanomedicine may offer some possible clinical alternatives.

Nanoparticles (NPs, with size ranging from 1 to 100 nm) are gaining prominence and emerging as the key basic components of nanotechnology due to the large number of applications including biomedicine, geology, chemistry, optics, catalysis, electronics and agriculture [[9], [10], [11], [12], [13], [14], [15], [16], [17]]. Generally, NPs can be produced through conventional physical and chemical methods [[15], [18], [19]]. More recently, green synthesis is becoming more popular as researchers look for new ways to make NPs. Green synthesis has many advantages over conventional methods because it can minimize NP toxicity and environmental implications by using environmentally-friendly and biodegradable biological sources [[9], [20], [21], [22]]

Plants have long been a rich source of therapeutic solutions for humans, both preventive and curative [23]. Globally around 35,000–70000 plant species are being used for therapeutic purposes [24]. They are a valuable source for the extraction of key bioactive components that may contribute to the emergence of novel drugs [25,[[26], [27], [28]]. The medicinal uses of plants is dependent on their intrinsic tissue phytochemistry. A good understanding of these phytochemicals contributes positively to a better understanding of plants' therapeutic potential [29,[[26], [27], [28], [30]]. Biochemical compounds can be obtained from different of the plant parts, such as roots, leaves, stem, bark, flowers, fruits and seeds [[26], [27], [28], [30], [31], [32],].

On the other hand, phyto-nanotechnology has recently opened new possibilities to synthesize nanoparticles (NPs). Biocompatibility scalability, and therapeutic potential against longstanding medical complications are all major benefits of phyto-nanotechnology [[33], [34]]. Metal nanoparticles are widely used in medical research [35]. Comparing it to other metal oxide nanoparticles, zinc oxide nanoparticles are cost-effective and exhibit minimum toxicity. These are considered to be exhibiting remarkable biomedical applications in the treatment of a various health issues, including cancer, bacterial inflammation, oxidative stress, diabetes, wounds and aids in drug delivery mechanism and bioimaging [36].

ZnO nanoparticles are generalized and more biocompatible, and the FDA has recognized the bulkier form of ZnO as a safe material [9]. Interestingly, ZnO nanoparticles proved to have selective cytotoxicity against the designated cancerous cell. The major process utilized by ZnO nanoparticles to induce cytotoxicity is by causing oxidative stress to the cancer cells [37,20].

Human system acquires Zinc (Zn) in traces and six enzyme classes contains Zn which includes isomerases, transferases, oxidoreductases, hydrolases, lyases and ligases [38]. Primarily compounds having Zn are potentially considered toxic for mammals and plants [39]. ZnO nanoparticles are generalized more biocompatible, and FDA have recognized bulkier form of ZnO as safe. ZnO nanoparticles administrated in the body can be degraded and take part in the nutritional cycle of the body [9,[40], [41], [42]]. Moreover, ZnO nanoparticles have been reported for their selective cytotoxicity against the designated cancerous cell, using the natural property they can also be surface engineered for increased selective cytotoxicity [43,44]. Extracellular administrations of ZnO nanoparticles (NPs) showed the biocompatibility but when these nanoparticles were administrated intracellularly at elevated level, they do not show biocompatibility but enhanced cytotoxicity using the oxidative stress and zinc-mediated protein activity disequilibrium [45]. The ZnO NPs coated with some polymeric material on the other hand is considered effective against wound healing, ulcers and a strong antimicrobial along with cancer treatment [46]. The coated ZnO NP decreases the expression of inflammatory cytokines by working against the NF- kB [47]. *Cassia* *fistula* based ZnO NPs have been shown to be effective antioxidant using DPPH assay [48]. Synthesizing ZnO NPs from *A. paniculata* leaf extracts has anti - oxidant, hypoglycemic, and anti-inflammatory properties, and may be used in a variety of biological applications such as cosmetology, food supplements and biomedical sectors [49].

*Acacia nilotica*, belonging of the Fabaceae family, is indigenously known as "Kikar” or “Babul” [50]. It is a multipurpose tree with a height of 5–18 m and is extensively distributed in subtropical and tropical regions of the world with about 1350 species. Traditional knowledge of *Acacia nilotica* reveals that all parts of the plant being used to treat diverse medical conditions. Pods and leaves of *Acacia nilotica* are reported to have potential antipyretic, antifungal, antibacterial, antimutagenic, antimalarial, anti-diabetic, anti-oxidative, and cytotoxic properties [51]. The bark and leaves of *Acacia nilotica* are a rich source of a wide variety of bioactive compounds, including steroids, alkaloids, saponins, terpenoids, tannins, and flavonoids. All these compounds have reported medicinal properties [52,53]. Numerous factors, such as the solvent selected for extraction, the technique used for extraction, drying temperature, storage duration, and environmental conditions, affect the quality and quantity of a phytochemical [54,[26]. Solubility, cost, selectivity, and safety should be taken into account [[27], [28]]. The ability of *Acacia nilotica* aqueous extracts as a bioreductant to produce nanoparticles from silver-doped TiO_2_, iron nanoparticles (FeNPs), ZnO-NPs and gold nanoparticles was reported; these have been evaluated for their potential as antimicrobial and anticancer agents in a number of studies [55,56].

This study presents a thorough investigation of the potential anticancer, antidiabetic, and antioxidant properties of ZnO nanoparticles (NPs) synthesized via a green approach; compared alongside with the solvent-based plant extracts. This work leverages upon the synergy between an environmentally-friendly green synthesis techniques and the therapeutic potential of natural plant products. Notably, this investigation stands amongst the first to explore the combined effects of ZnO NPs and plant extracts on treating cancer and diabetes, while also evaluating their antioxidant capacity. By elucidating the multifaceted bioactivities of these innovative nanocomposites, our research not only advances the field of green nanotechnology but also provides valuable insights for the development of promising therapeutic candidates against cancer and diabetes.

## 2 Material and methods

### 2.1 Preparation of plant extract from *Acacia nilotica*

#### 2.1.1 Preparation of aqueous extracts for ZnO nanoparticles

*Acacia nilotica* bark samples of a single genotype were obtained from Ayub Agriculture Research Institute, Faisalabad, Pakistan. The departmental herbarium identified the samples. The aqueous extract derived from both the bark and leaves of *Acacia nilotica* was formulated by dissolving 10g of bark material in 100 mL of deionized water [57]. Prior to the synthesis of plant extract, the barks were washed three times with distilled water and subjected to drying at room temperature. Fine-powdered form of bark was obtained through proper grinding. The prepared extract was heated for 15 min at 50 °C and placed in a shaking incubator for 24h for the extraction of polyphenols, followed by the addition of 50 mM H_3_PO_4_. For the filtration process, the extract was passed through Whatman no. 1 filter paper and the resulting filtrate was kept at 4 °C to carry out further experiments.

#### 2.1.2 Methanol and ethanol-based plant extracts

Methanol and ethanol-based plant extracts were prepared using the method following the method outlined by Ref. [58] A fine powdered form of *Acacia nilotica* bark and leaves was dissolved in ethanol and methanol at room temperature, i.e., 1 g of each sample was dissolved in 10 mL ethanol and methanol, followed by constant shaking for 24h. The filtered solvents were left to evaporate at room temperature until complete solvent removal was achieved. The obtained dried extracts were further dissolved in an equivalent volume of Phosphate buffer saline. Different dilutions were prepared from 0.1 μg/mL, 1 μg/mL, 10 μg/mL, 100 μg/mL, and 1000 μg/mL for further investigation.

### 2.2 The green synthesis of ZnO nanoparticles using *Acacia nilotica*

To fabricate the Zinc Oxide Nanoparticles (ZnO-NPs) from *Acacia nilotica*, a solution of zinc nitrate salt was utilized as the precursor. Aqueous plant extract (10 mL) was dissolved into a ZnO salt solution upon constant stirring under a dark environment for approximately 4h. A color change in the solution validates the synthesis of ZnO-NPs. The reaction mixture underwent centrifugation for a duration of 30 min at 13000 rpm. The obtained pellet was further subjected to continuous washing for the removal of debris and impurities. The solution was then passed from Whatman no 1 filter paper, keeping the pallet dispersed. After drying at 70 °C for 24 h, the obtained product underwent calcination, leading to the formation of a pure powder form of ZnO nanoparticles (ZnO-NPs). This powder was subsequently stored for further analysis.

### 2.3 The characterization of ZnO-NPs

A wide range of characterization techniques were applied to the synthesized ZnO-NPs, including UV–Vis spectroscopy, FTIR, and SEM analysis. UV–Visible spectrophotometer was used for the analysis of the absorbance spectrum of ZnO-NPs, keeping the range from 200 nm to 800 nm. A similar range was used previously by Ref. [59]. Furthermore, the physical properties of ZnO-NPs, including shape, size, and morphological directions, were obtained through SEM analysis. A scanning electron microscope was used for microscopic analysis of ZnO-NPs using double-sided carbon tape. The coverslip was fixed to the aluminum stub after cooling. Zinc nanoparticles solution was then poured onto the slide and dried at 100 °C on the hot plate [60]. For FTIR analysis, the sample was placed in an IR source path where the beams were split into two equal beams of the same intensity. The detector was used for the conversion of signals into the spectrum. A similar procedure was reported by Ref. [61] for FTIR analysis. Signals and beams were identified by the pictorial representation of data on a computer, and the results were generated using OriginLab software. To undertake XRD analysis, a drop of ZnO-NPs sample was dropped onto the glass substrate. It was then kept in an X-ray diffractometer at 45 kV, 40 mA, with Zn-Kα rays. A manual analysis of structural crystallinity was conducted. In order to calculate the average particle size, Debye-Scherrer's formula, suggested earlier by Ref. [62], was utilized.

### 2.4 Evaluation of anti-cancer activity-MTT assay

For evaluating the anti-cancer potential of nanoparticles, ethanol/methanol-based plant extracts and cells were first cultured in Dulbecco's modified Eagle's medium (DMEM). The cells were placed in 75 cm^2^ culture flask containing 10 mL DMEM. To improve cells growth, 10 % fetal bovine serum (FBS) along with 1 % 100x l-Glutamine was also added to the culture flask, keeping the temperature 37 °C and CO2 concentration to 5 %. Detachment of the cells from the base of the flask was proceeded by the addition of 2–5 mL trypsin. Single-cell suspension produced by the detached cells was further transferred into another flask containing 10 mL DMEM, where reattachment and division of the cells were initiated. The cells were further utilized for MTT assay trials.

For evaluation of anti-cancer activity, an MTT assay was performed on HepG2 liver cancer cell lines in 96- well plates following the methodology mentioned earlier by Refs. [57,60,63] along with slight modifications. The cells were seeded into the wells and maintained at optimum temperature (37 °C) in the presence of 5 % CO_2_ overnight. In each well, 3 μL of ZnO-NPs and plant extracts were added, and subsequently, the plate was placed in an incubator for a duration of 48 h. Another incubation was given for 4h after the addition of 10 μL MTT (3-(4,5- dimethylthiazol-2-yl)-2,5-diphenyltetrazolium bromide). After 4h, dimethyl sulfoxide was added, and the plate was incubated again for 30 min. The OD was taken using an ELISA plate reader at 630 nm for the assessment of cell death. The obtained data were analyzed statistically using GraphPad Prism 2021 and MS Excel 2016. The below-mentioned equation (equation (1)) was used for the estimation of inhibition percentage.

(Equation 1) Inhibition percentage = Ac — As / Ac × 100

Where, As = absorbance of treated cells, Ac = absorbance of untreated cells.

### 2.5 Evaluation of anti-oxidant activity-DPPH assay

The anti-oxidant potential of ZnO-NPs and ethanol/methanol-based plant extracts was evaluated using a DPPH assay, adopting the methodology described by Refs. [60,63] with slight amendments. Gallic acid (3 mM) was used as a positive control. 10 mL ZnO-NPS and plant extracts were added to each well of the 96-well plate, followed by the addition of 3 Mm DPPH in a dark environment. The plate was incubated for 30 min in an incubator, and a change in color was observed. The OD was obtained using an ELISA plate reader at 490 nm. The obtained data were analyzed statistically using GraphPad Prism 2021 and MS Excel 2016. The inhibition % was calculated using above mentioned Equation (1).

### 2.6 Evaluation of anti-diabetic activity-- α-glucosidase assay

The anti-diabetic potential of ZnO-NPs and ethanol/methanol-based plant extracts were evaluated using α-glucosidase assay, adopting the methodology described by Ref. [58] with slight amendments. Acarbose (a clinically effective drug against diabetes) was utilized as a positive control, whereas for negative control, PBS was used. 12.5 μL ZnO-NPs and plant extracts were added to the 96-well plate along with the addition of 40 μL (0.5 units/mL) α-glucosidase enzyme. 140 μL of PBS was added into each well, followed by incubating the plate for approximately 5 min. After incubation, 40 μL PNPG (5 mM) substrate was added, and the plate was kept in an incubator for 30 min. The absorbance of the samples was evaluated using an ELISA plate reader at 405 nm. The obtained data were analyzed statistically using GraphPad Prism 2021 and MS Excel 2016. The inhibition % was calculated using Equation 1.

## 3 Results and discussion

### 3.1 Biosynthesis of ZnO-NPs/preparation of ethanol and methanol based plant extracts

The bark and leave extracts of *A. nilotica* are reported to possess several medicinal properties, including anti-cancer, anti-diabetic and anti-oxidative potential, due to which it is a center of attention for scientists and used by numerous researchers including [[64], [65], [66], [67]] in their studies which showed the promising results of this plant against the aforementioned complications. The bark extract of *A. nilotica* was used for the preparation of zinc oxide nanoparticles that showed the potential of the plant for the biosynthesis of nanoparticles. The color change of nanoparticles was observed from light-yellow to deep brownish-yellow, corresponding to the findings of [68], which confirmed the biosynthesis of NPs. Alongside that, solvent-based extracts were also prepared in which ethanol and methanol were used as a solvent. Similar solvents were used by multiple researchers to achieve polyphenol rich, including [58,69,70]. [69] indicated that compared to water, ethanol and methanol are better solvents for plant extractions. However, the fluctuation in results between ethanol and methanol was also reported in different studies. Ethanol was reported as a more effective solvent for the extraction of plants as compared to methanol [58,71]. The current study further investigates and provides the comparative analysis of zinc nanoparticles and solved-based plant extracts against cancer, diabetes, and oxidative stress.

### 3.2 The UV–Vis spectrophotometric analysis of ZnO-NPs

**Fig. 1:**
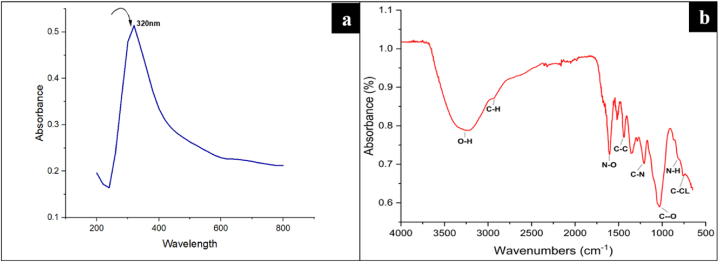
**(a)** UV–visible spectrophotometric Analysis of ZnO-NPs, **(b)** Analysis of functional-groups location of ZnO-NPs using FTIR spectroscopy.

The synthesized ZnO-NPs were subjected to UV–visible spectrophotometric analysis. Maximum absorbance of nanoparticles was observed at 320 nm (Fig. 1A), corresponding to the findings of [37,49,72], which confirmed the synthesis of ZnO-NPs.

### 3.3 The FTIR analysis of ZnO-NPs

FTIR spectroscopy was used to locate functional groups in *Acacia nilotica* that contribute to ZnO-NP production. The FTIR spectra analysis of ZnO-NPs indicated an absorption band at 2924.08 cm-1, suggesting the presence of C–H stretching in an alkane group. Phenolic compounds were confirmed by the O–H stretching observed at 3263.55 cm-1 and the C–O peak at 1020.05 cm-1. Additionally, the absorption band at 1213.22 cm-1 revealed the presence of the C–N functional group, and the N–H bend was observed at 815.88 cm-1 in ZnO-NPs, which confirms the presence of amines (Fig. 1B). Other functional groups have been shown in the FTIR spectra along with phenolics and amines. These functional groups are found in a variety of phytochemicals that induces the reduction of ZnO, including alkaloids and amines, seed oils, fatty acids, non-protein amino acids, gums, cyclitols, fluoroacetate, hydrolyzable tannins and terpenes including triterpene genins, diterpenes, saponins, phytosterol, and essential oils. Most of the functional groups obtained in the results of ZnO are similar to those reported previously by Refs. [68,73], whereas; the variation in other functional groups and detected findings is due to the nature of plant which differs from the previously published studies.

### 3.4 The SEM analysis of ZnO-NPs

**Fig. 2:**
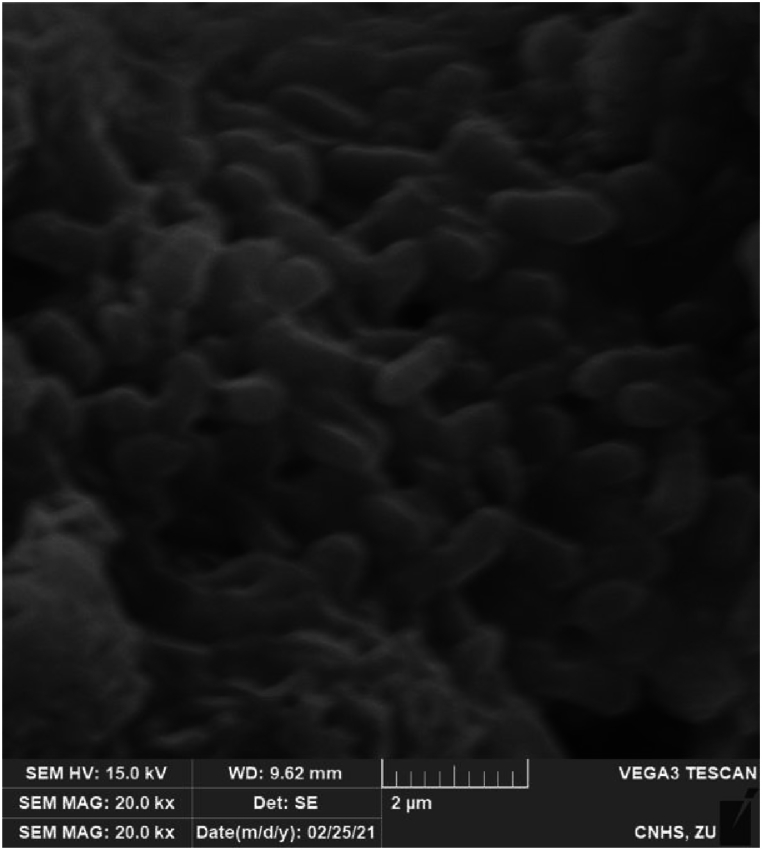
Scanning Electron Microscopic images of Zinc nanoparticles fabricated using bark extract of *Acacia nilotica*.

Physical characteristics of the synthesized ZnO-NPs, including shape or morphology, were identified using a scanning electron microscope (FEI brand model Inspect S50). The majority of nanoparticles were determined to be triangular in shape (as shown in Fig. 2). A similar shape of zinc oxide nanoparticles was observed earlier by Ref. [74]. No agglomeration was seen, which reflects the formation of purified nanoparticles. Different factors influence the morphological parameters of nanoparticles, including plant, salt concentration, salinity stress, and PH [75,76]. The fluctuation in PH, in the case of plants, alters the charges of natural phytochemicals, which in turn affects the binding ability and metal ions reduction during the synthesis of nanoparticles, ultimately affecting its morphology and yield [76].

### 3.5 The XRD analysis of ZnO-NPs

**Fig. 3:**
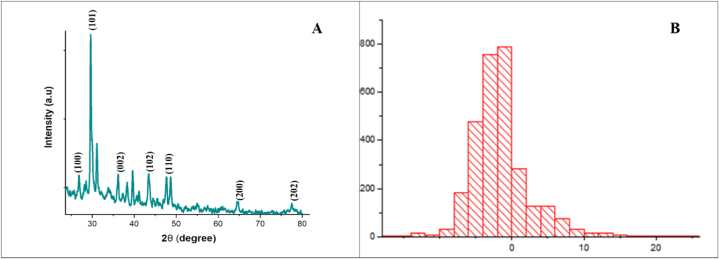
**(a)** X-ray diffraction analysis of ZnO-NPs synthesizes from *Acacia nilotica,***(b)** ZnO-NPs average size of particles: estimated to be (15 ± 1.5).

The XRD pattern provided relevant information about the changes in diffraction peaks' positions that were used to make conclusions about cell parameters and crystalline structure due to changes in nanoparticle size and shape [77]. X-ray diffraction analysis of ZnO-NPs in the current study clearly indicates the crystalline structure of ZnO-NPs. Prominent and sharp diffractions peaks were observed at 25.22, 30.01, 37.11, 43.98, 64.09, and 78.53° (2θ values) indexed as (100), (101), (002), (102), (110), (200) and (202) diffraction lattice planes as shown in (Fig. 3A). The maximum peak was observed at (101), corresponding to the findings of [78]. The obtained diffraction peaks confirm the crystallinity of synthesized ZnO-NPs. The mean particle size of ZnO-NPs was calculated using Debye–Scherer equation (D = Kωβcosθ) (Fig. 3B). The average particle size obtained was (15 ± 1.5). A similar range of particle size was reported earlier by Refs. [79,80], supporting the findings of the current study.

### 3.6 Comparative analysis of ZnO-NPs and ethanol/methanol-based plant extracts

#### 3.6.1 Evaluation of the anti-cancer activity—MTT assay

**Fig. 4:**
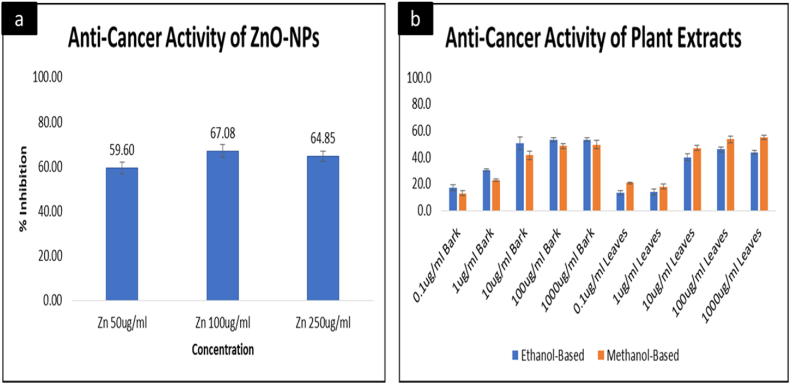
Comparison between the anti-cancer activity of Zinc oxide nanoparticles and solvent-based plant extracts, 4a)-the anti-cancer activity of ZnO-NPs, 4b) - the anti-cancer activity of ethanol and methanol-based plant extracts using barks and leaves of *Acacia nilotica*. Values are presented as mean ± SD of three different experiments. Analysis of variance (ANOVA) reveals the significant influence of tested groups; p < 0.0001.

Liver cancer is a major health challenge worldwide that needs effective and advanced therapeutic research as it poses a significant diagnostic and prognostic challenge for clinicians [81]. The practice of nanotechnology could enhance the efficacy and selectivity of physical, chemical, and biological approaches to target cancer cells while reducing collateral damage to nonmalignant cells. Alongside that, bioactive compounds, polyphenols, and plant-based extracts plays central role in treating number of diseases including liver cancer. MTT assay was performed to examine the anti-cancer potential of nanoparticles in comparison with the solvent-based extracts of *Acacia nilotica* against HepG2 cancer cell lines. The ZnO-NPs were prepared at 50 μg/mL, 100 μg/mL, and 250 μg/mL, respectively. The obtained results (Fig. 4a) indicated that the maximum inhibition of cancer cells was achieved at 100 μg/mL. Similarly, the different concentrations of solvent-based extracts of leaves and barks of *Acacia nilotica* were also tested. For that purpose, the extracts were prepared at five different dilutions i.e., 0.1 μg/mL, 1 μg/mL, 10 μg/mL, 100 μg/mL, and 1000 μg/mL (Fig. 4b). is the visual representation of obtained results indicating that no significant difference between the activities leaves and barks were seen, but a minor difference was observed in a dose-dependent manner from 0.1 μg/mL to 1 μg/mL. The percentage of inhibition increased at 10 μg/mL in the case of bark extract, whereas the extract prepared from leaves showed no significant difference from 0.1 μg/mL to 1 μg/mL but presented a visible difference in concentrations ranging from 10 μg/mL to 1000 μg/mL. Both leaves and bark-based extracts showed different results on different solvents (Ethanol and methanol). Ethanol showed better results for bark-based plant extract, whereas, in the case of leaves-based extract, methanol proved to be a better solvent, reflecting that both ethanol and methanol act as effective solvents, depending upon different parts of selected sources of polyphenols. In order to extract polyphenols from their natural environments, ethanol and methanol are considered the most efficient solvents, as suggested by Refs. [52,82,83]. Some researchers, including [58,84], concluded ethanol as a better solvent for plant extraction, whereas [85], suggested that methanol serves as a better solvent for polyphenol extraction. A possible explanation for this variation is the plant parts that are sampled and the geographical location from where the samples were collected [86]. Another possible reason reflecting the better cytotoxic activity of methanol could be the presence of gallocatechin 5-O gallate, a cytotoxic compound reported to be present in methanol extracts of *Acacia nilotica* by Ref. [87]. The current study provided a comparative analysis of the anti-cancer potential of solvent-based extracts and zinc oxide nanoparticles using the same plant. According to Fig. 4a and Fig. b, ZnO-NPs are comparatively more efficient and constitute higher potential against HepG2 cell lines than solvent-based plant extracts. Nanoparticles being smaller in size, reaches target positions in cells more efficiently, and ZnO-NPs lead to the death of cancerous cells without causing cytotoxicity to normal cells [12]. In parallel to another study [88], it was revealed that the zinc oxide NPs induced apoptosis, DNA damage, and decreased cell viability in HepG2 cancer cell lines, supporting the acquired results of our study.

#### 3.6.2 Evaluation of the anti-oxidant activity-DPPH assay

**Fig. 5:**
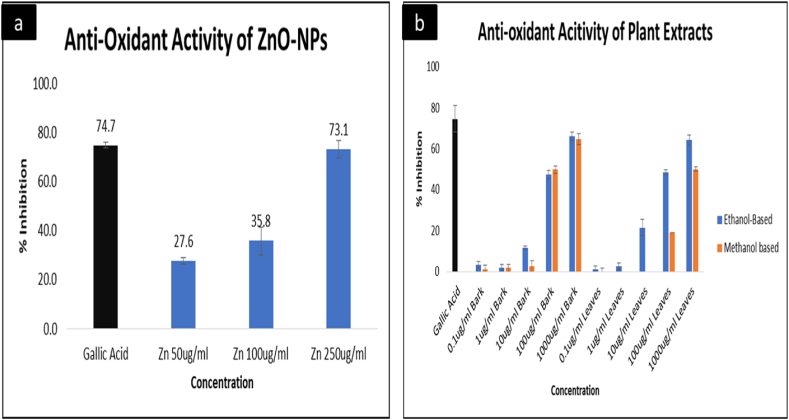
Comparison between the anti-oxidant activity of Zinc oxide nanoparticles and solvent-based plant extracts, 5a)-the anti-oxidant activity of ZnO-NPs, 5b) - the anti-oxidant activity of ethanol and methanol-based plant extracts using barks and leaves of *Acacia nilotica*. Values are presented as mean ± SD of three different experiments. Analysis of variance (ANOVA) reveals the significant influence of tested groups; p < 0.0001.

Oxidative stress is linked with the development of numerous metabolic disturbances and chronic diseases, including cancer and diabetes. When pro-oxidants are exposed for the long term, they may cause structural and functional changes to mitochondrial DNA, along with modifications in cell structures and enzymes, leading to gene expression irregularities [89]. Herbal medicine and Phyto-nanotechnology play crucial role in the management of oxidative stress in the body. DPPH assay was performed to investigate the anti-oxidative potential of ZnO-NPs in comparison with the solvent-based extracts of *Acacia nilotica*. The ZnO-NPs were prepared at 50 μg/mL, 100 μg/mL, and 250 μg/mL, respectively. Gallic acid was utilized as a positive control due to its proven radical scavenging properties, as reported previously by Refs. [60,90,91]. The obtained results (Fig. 5a) demonstrated that maximum inhibition was achieved at the highest concentration, i.e., 250 μg/mL. However, there was a slight difference in inhibition activities obtained at 50 μg/mL and 100 μg/mL. A sudden increase in inhibition percentage was observed at 250 μg/mL, demonstrating the fact that increasing the concentration of ZnO-NPs increases the radical scavenging properties. However, a dose-dependent manner was observed in the results, corresponding to the findings of [48,92], reflecting that ZnO-NPs at 250 μg/mL constitute the highest potential to reduce oxidative stress. On the other hand, the solvent-based extracts were prepared from both leaves and barks of *Acacia nilotica*. For that purpose, the extracts were prepared at five different dilutions i.e. 0.1 μg/mL, 1 μg/mL, 10 μg/mL, 100 μg/mL, and 1000 μg/mL, respectively. In the case of bark extracts, dose-dependent activities of both ethanol and methanol were observed. The results did not differ significantly from 0.1 μg/mL to 1 μg/mL, and the activity was extremely low. However, a slight increase in ethanol-based bark extracts were observed at 10 μg/mL, and the eventual increase were observed at 100 μg/mL and 1000 μg/mL providing maximum inhibition at 1000 μg/mL. In contrast, the extracts prepared from leaves of *Acacia nilotica* showed no activities from 0.1 μg/mL to 10 μg/mL but performed considerably better at 100 μg/mL and 1000 μg/mL in methanol. In contrast, a dose-dependent manner was observed in the case of ethanol, i.e., the inhibition activity was increased with the increase in dosage, concluding ethanol as a better solvent for anti-oxidant activity. One of the possible reasons for the enhanced scavenging activity of ethanol-based extracts could be its hydrogen-donating or electron-scavenging properties, which lead to the better anti-oxidant function of the ethanol extract, making it a potential source of anti-oxidants as described by Ref. [93] (Fig. 5a and b). provided another comparison between the anti-oxidative potential of ZnO-NPs and solvent-based extracts, which shows ZnO-NPs are more efficient and suitable agents for reducing oxidative stress, which can be supported by the fact that zinc itself provides protection to the cells against oxidative damage and plays an critical role in membrane stabilization by inhibiting NADPH oxidase as reported by Ref. [94].

#### 3.6.3 Evaluation of the anti-diabetic activity-- α-glucosidase assay

**Fig. 6:**
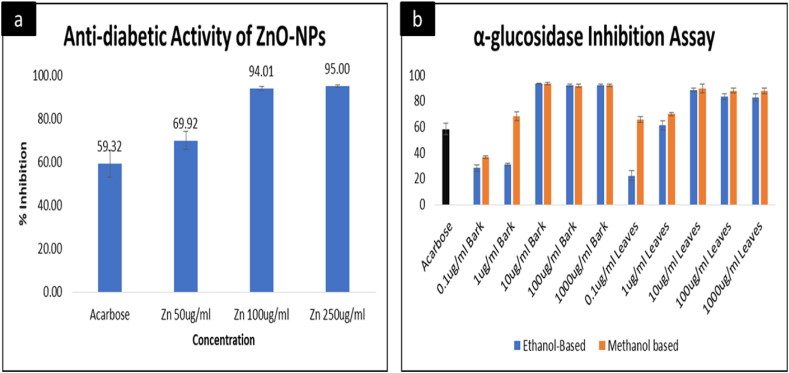
Comparison between the anti-diabetic activity of Zinc oxide nanoparticles and solvent-based plant extracts, 6a)-the anti-diabetic activity of ZnO-NPs, 6b) - the anti-diabetic activity of ethanol and methanol-based plant extracts using barks and leaves of *Acacia nilotica*. Values are presented as mean ± SD of three different experiments. Analysis of variance (ANOVA) reveals the significant influence of tested groups; p < 0.0001.

Diabetes has always been the major health challenge that makes it the most important research area. Despite numerous researches on diabetes, still, the lack of new anti-diabetic agents is a major concern, leading to the need for further investigations [95]. Polyphenols and plant-mediated nanoparticles exhibit remarkable anti-diabetic properties and are used by numerous researchers for their applications against multiple health complications [57,96]. The α-Glucosidase assay was performed to determine the anti-diabetic potential of zinc oxide nanoparticles in comparison with the solvent-based extracts of *Acacia nilotica*. The ZnO-NPs were prepared at 50 μg/mL, 100 μg/mL, and 250 μg/mL, and Acarbose was used as a positive control due to its proven anti-diabetic activity, as suggested by a number of researchers, including [58,60]. The results (Fig. 6a) demonstrated that the maximum inhibition was achieved at the highest concentration, i.e., 250 μg/mL. However, there is no significant difference between the results obtained at 100 μg/mL and 250 μg/mL, but a sudden drop was observed at 50 μg/mL, proving that zinc nanoparticles prepared from *Acacia nilotica* serve to be a promising agent in the treatment of diabetes at high concentrations. Moreover, a dose-dependent manner was observed, indicating that increasing the dose ultimately increases the activity of ZnO-NPs. A similar pattern was observed previously by Refs. [97,98], supporting the findings of the current study. On the other hand, the solvent-based extracts were prepared from both leaves and barks of *Acacia nilotica*. For that purpose, the extracts were prepared at five different dilutions i.e. 0.1 μg/mL, 1 μg/mL, 10 μg/mL, 100 μg/mL, and 1000 μg/mL, respectively. The concentration-dependent increase was observed in the anti-diabetic activity of both leaves and bark in ethanol and methanol. Overall, methanol is seen as a better solvent for the extraction of leaves and barks of *Acacia nilotica*. However, a difference between the inhibition activities of bark and the leaves-based extract was observed. According to (Fig. 6a and Fig. b), a non-significant variation in results can be seen from 10 μg/mL to 1000 μg/mL, but a sudden drop in inhibitory activities of barks and leaves were observed at 1 μg/mL and 0.1 μg/mL, indicating that both nanoparticles and solvent-based extracts from *Acacia nilotica* provide better results at higher concentrations in comparison to the low concentrations (Fig. 6a and Fig. b). also shows a comparison between the results obtained from ZnO-NPs and solvent-based extracts demonstrating ZnO-NPs as better inhibitors of α-Glucosidase, showing maximum inhibition (95 %) at the highest concentration applied, i.e., 250 μg/mL, whereas the maximum inhibition obtained by solvent-based extracts was 93 % at 10 μg/mL. According to Ref. [99], nanoparticles possess enhanced targeting ability, improved efficacy, and safety as compared to traditional methods. Improved efficacy and targeting ability of ZnO-NPs could be one of the major reasons supporting the better results of ZnO-NPs compared to conventional methods such as the utilization of solvent-based extracts. Alongside that [100],suggested that zinc plays a vital role in insulin secretion and storage, which could be another factor for increased efficacy of ZnO-NPs against diabetes, supporting the findings of the current study.

## 4 Conclusion

Nanotechnology could enhance the efficacy of various biological approaches to target different ailments in humans due to the unique molecular characteristics of nanoparticles. The results obtained from the ZnO-NPs, and solvent-based extracts were compared in this study; concluding that ZnO-NPS was a promising therapeutic agent against cancer, diabetes, and oxidative stress. The biogenic ZnO-NPs delivered better results than plant extracts which might be attributed to the ability of ZnO-NPs to traverse through the biological barriers of cells, interacting with cell components and causing apoptosis in cancer cells. In addition, nanoparticles might have restricted the angiogenesis: an essential step in tumor growth. For the first time, the comparative analyses between zinc oxide nanoparticles and solvent-based extracts using two different solvents (ethanol and methanol), and two different plant parts (barks and leaves), were reported for the first time. These observations provided deep insights into the efficacy and reliability of ZnO-NPs in comparison to the conventional methods. Further investigation is warranted with regards to the use of green synthesized ZnO nanoparticles for *in vivo* study. This proposed study might lead to the development of novel nanoparticles-based anticancer medicine.

## Data availability statement

All of the data are available in all Tables and Figures of the manuscripts.

## CRediT authorship contribution statement

**Muhammad Azeem:** Writing – original draft, Methodology, Formal analysis, Data curation. **Muhammad Hussnain Siddique:** Writing – review & editing, Visualization, Validation. **Muhammad Imran:** Writing – review & editing, Resources, Formal analysis. **Muhammad Zubair:** Writing – review & editing, Project administration, Funding acquisition, Conceptualization. **Rabia Mumtaz:** Writing – review & editing, Validation, Data curation. **Madiha Younas:** Writing – review & editing, Formal analysis, Data curation. **Mostafa A. Abdel-Maksoud:** Writing – review & editing, Funding acquisition. **Mohamed A. El-Tayeb:** Writing – review & editing, Validation. **Muhammad Rizwan:** Writing – review & editing, Software. **Jean Wan Hong Yong:** Writing – review & editing, Software, Funding acquisition.

## Declaration of competing interest

The authors declare that they have no known competing financial interests or personal relationships that could have appeared to influence the work reported in this paper.
