# Supplementary material for: Assessing anticancer, antidiabetic, and antioxidant capacities in green-synthesized zinc oxide nanoparticles and solvent-based plant extracts
Source: Heliyon. 2024 Jul 4;10(14):e34073. doi: 10.1016/j.heliyon.2024.e34073 (PMC11292250; doi:10.1016/j.heliyon.2024.e34073)
Supplement: Multimedia component 1 [file mmc1.docx]

| **Species** | **Tissues used for Extraction** | **Types of NPs** | **Shape** | **Size (nm)** | **Application** | **Refs.** |
| --- | --- | --- | --- | --- | --- | --- |
| *Sargassum algae* | Algal | Palladium(Pd) | Octahedral | 4-10 | Electrolytic activities | **[1]** |
| *Panax*  *ginseng* | Roots | Gold(Au) & silver(Ag) | Spherical | Gold is 15-40; Silver is 10-25 | Anti-bacterial | **[2]** |
| *Ginkgo biloba* | Leaves | Copper | Spherical | 10-20 | Catalytic | **[3]** |
| Banana | Peel(skin) | Cadmium-Sulfide |  | 1.47 | Catalytic Activity | **[4]** |
| *Artocarpus gomezianus* | Fruits | Zinc(Zn) | Spherical | >18 | Fluorescence | **[5]** |
| Orange and pineapple | Fruit | Silver(Ag) | Spherical | 10-250 |  | **[6]** |
| *Gardenia jasminoides* | Leaves | Iron(Fe) | physical appearance like rock | 30 | Anti-bacterial | **[7]** |
| *Catharanthus roseus* | Leaves | Palladium(Pb) | Spherical | 35 | degradation of dye catalytically | **[8]** |
| *Euphorbia prostrata* | Leaves | Titanium dioxide(TiO_2)_ | Spherical | 81-84 | Leishmanicidal | **[9]** |

**Supplementary Table 1.** Tissue used for synthesis of biogenic nanoparticles, size, shape, type and their biological activities

1. Momeni, S. and I. Nabipour, *A simple green synthesis of palladium nanoparticles with Sargassum alga and their electrocatalytic activities towards hydrogen peroxide.* Applied biochemistry and biotechnology, 2015. **176**: p. 1937-1949.

2. Singh, P., et al., *The development of a green approach for the biosynthesis of silver and gold nanoparticles by using Panax ginseng root extract, and their biological applications.* Artificial cells, nanomedicine, and biotechnology, 2016. **44**(4): p. 1150-1157.

3. Nasrollahzadeh, M. and S.M. Sajadi, *Green synthesis of copper nanoparticles using Ginkgo biloba L. leaf extract and their catalytic activity for the Huisgen [3+ 2] cycloaddition of azides and alkynes at room temperature.* Journal of Colloid and Interface Science, 2015. **457**: p. 141-147.

4. Zhou, G.J., et al., *Biosynthesis of CdS nanoparticles in banana peel extract.* Journal of nanoscience and nanotechnology, 2014. **14**(6): p. 4437-4442.

5. Suresh, D., et al., *Artocarpus gomezianus aided green synthesis of ZnO nanoparticles: Luminescence, photocatalytic and antioxidant properties.* Spectrochimica Acta Part A: Molecular and Biomolecular Spectroscopy, 2015. **141**: p. 128-134.

6. Hyllested, J.Æ., et al., *Green preparation and spectroscopic characterization of plasmonic silver nanoparticles using fruits as reducing agents.* Beilstein Journal of Nanotechnology, 2015. **6**(1): p. 293-299.

7. Naseem, T. and M.A. Farrukh, *Antibacterial activity of green synthesis of iron nanoparticles using Lawsonia inermis and Gardenia jasminoides leaves extract.* Journal of Chemistry, 2015. **2015**.

8. Kalaiselvi, A., et al., *Synthesis and characterization of palladium nanoparticles using Catharanthus roseus leaf extract and its application in the photo-catalytic degradation.* Spectrochimica Acta Part A: Molecular and Biomolecular Spectroscopy, 2015. **135**: p. 116-119.

9. Zahir, A.A., et al., *Green synthesis of silver and titanium dioxide nanoparticles using Euphorbia prostrata extract shows shift from apoptosis to G0/G1 arrest followed by necrotic cell death in Leishmania donovani.* Antimicrobial agents and chemotherapy, 2015. **59**(8): p. 4782-4799.
